# Growing coral larger and faster: micro-colony-fusion as a strategy for accelerating coral cover

**DOI:** 10.7717/peerj.1313

**Published:** 2015-10-20

**Authors:** Zac H. Forsman, Christopher A. Page, Robert J. Toonen, David Vaughan

**Affiliations:** 1 Division of Aquatic Resources, State of Hawaii, Honolulu, HI, United States; 2 Hawaii Institute of Marine Biology, Kaneohe, HI, United States; 3 Mote Marine Laboratory, Sarasota, FL, United States; 4 Mote Marine Laboratory, Summerland Key, FL, United States

**Keywords:** Coral cultivation, Colony fusion, Colony fragmentation, Coral restoration, Coral growth, *Porites lobata*, *Orbicella faveolata*, *Pseudodiploria clivosa*

## Abstract

Fusion is an important life history strategy for clonal organisms to increase access to shared resources, to compete for space, and to recover from disturbance. For reef building corals, fragmentation and colony fusion are key components of resilience to disturbance. Observations of small fragments spreading tissue and fusing over artificial substrates prompted experiments aimed at further characterizing Atlantic and Pacific corals under various conditions. Small (∼1–3 cm^2^) fragments from the same colony spaced regularly over ceramic tiles resulted in spreading at rapid rates (e.g., tens of square centimeters per month) followed by isogenic fusion. Using this strategy, we demonstrate growth, in terms of area encrusted and covered by living tissue, of *Orbicella faveolata*, *Pseudodiploria clivosa*, and *Porites lobata* as high as 63, 48, and 23 cm^2^ per month respectively. We found a relationship between starting and ending size of fragments, with larger fragments growing at a faster rate. *Porites lobata* showed significant tank effects on rates of tissue spreading indicating sensitivity to biotic and abiotic factors. The tendency of small coral fragments to encrust and fuse over a variety of surfaces can be exploited for a variety of applications such as coral cultivation, assays for coral growth, and reef restoration.

## Introduction

For many organisms, size is closely correlated to survivorship, fecundity, and outcome of competitive interactions (e.g., Hughes, Ayre & Connell, 1992; Hall & Hughes, 1996; Sakai, 1998; Smith & Hughes, 1999; Palmer, 2004; Ayre & Grosberg, 2005; Marshall, Cook & Emlet, 2006). For clonal organisms such as corals, the smallest size classes (e.g., including larvae, newly settled recruits, and small fragments) suffer the highest rates of mortality (Vermeij & Bak, 2002; Raymundo & Maypa, 2004; Forsman, Rinkevich & Hunter, 2006). Coral colonies above a size threshold shift resources from growth to sexual reproduction (Babcock, 1991; Soong, 1993; Kojis & Quinn, 2001). Likewise, when a sexually mature colony is fragmented below a certain size, resources are allocated towards regrowth instead of reproduction (Lirman, 2000; Zakai, Levy & Chadwick-Furman, 2000). Fragmentation and fission (division of the colony) are common for reef building corals, resulting from factors such as: physical disturbance (Brandt et al., 2013), wave damage (Dollar, 1982), erosion, predation (e.g., from parrot fish bites Boulay et al., 2014), sedimentation (Nugues & Roberts, 2003), disease, parasitism (Gochfeld & Aeby, 1997), and partial bleaching (Schuhmacher et al., 2005; Roff et al., 2014). In contrast, fusion (portions of a colony growing together) can be an important strategy for small coral colonies to grow as quickly as possible for a number of reasons including; (1) more access to shared resources, (2) a competitive advantage from occupying more space, (3) regaining sexual maturity and reproductive capacity (Okubo, Motokawa & Omori, 2006), and (4) escaping vulnerability associated with small colony size (Raymundo & Maypa, 2004; see Maldonado, 1998 for a counter example in sponges; reviewed by Grosberg, 1988). Fusion may occur between genetically identical fragments, or from larvae that settle gregariously (Raymundo & Maypa, 2004). Juvenile cnidarians may also fuse with kin (Grosberg & Quinn, 1986; Wilson & Grosberg, 2004), conspecifics, and possibly even congeners, resulting in chimerism (Rinkevich, 2004; Work et al., 2011). Chimerism (fusion between genetically distinct colonies) has generally been associated with conflicts among the partners (Puill-Stephan et al., 2009; Puill-Stephan et al., 2012; Work et al., 2011); however, it has also been shown to confer benefits for some clonal organisms by allowing expression of alternative phenotypes in contrasting environments (Rinkevich & Weissman, 1992; Pineda-Krch & Lehtilä, 2004). However, the present study focuses exclusively isogenic fusion, which is defined as the fusion of several ramets (fragments) from the same genet (parent colony).

Previous experimental work with corals has shown that fusion can reduce size specific mortality among juvenile corals (Raymundo & Maypa, 2004), and controlled conditions can increase survivorship of small colonies (Raymundo & Maypa, 2004; Forsman, Rinkevich & Hunter, 2006; Toh et al., 2013). Culture of juvenile colonies or small (e.g., ∼1 cm^2^) fragments combined with fusion of genetically identical colonies (micro-colony fusion) is a potential growth enhancement strategy for coral aquaculture. The ability to promote rapid growth over a pre-determined substrate would be a beneficial tool for a range of applications such as propagation of rare coral species, for the development of standardized growth assays, coral aquaculture, and reef restoration. We examined fusion in *Orbicella faveolata* and *Pseudodiploria clivosa* to quantify rates of area increase. Similarly, we conducted an experiment with *Porites lobata* to characterize tissue spreading and to determine if the rates are influenced by biotic and abiotic factors in two contrasting tank environments. In addition, we compile both qualitative and quantitative examples of isogenic colony fusion across a variety of coral species in both the Atlantic and Pacific Oceans.

## Methods

### *Orbicella faveolata* and *Pseudodiploria clivosa* fusion experiments

**Figure 1:**
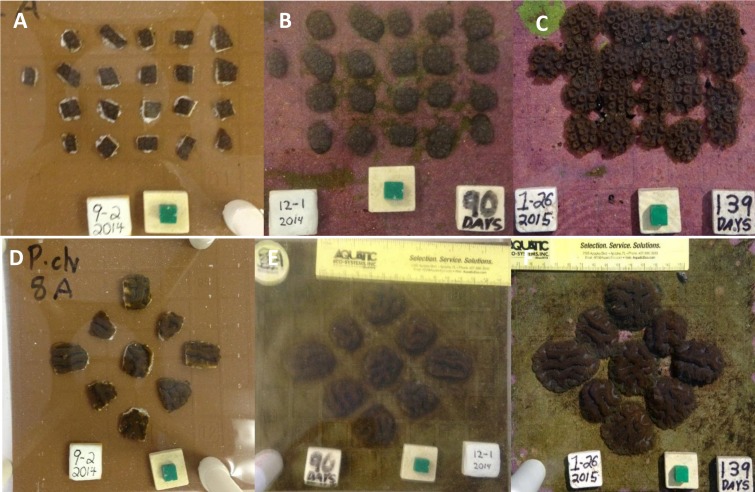
*Orbicella faveolata* and *Pseudodiploria clivosa* fusion experiments. (A) Initial fragments of *O. faveolata*; (B) the fragmented colonies after 90 days; (C) the fragmented colonies at 139 days as colonies begin to fuse; (D) initial fragments of *P. clivosa*; (E) the same fragmented colonies after 90 days; (F) the fragmented colonies after 139 days as fusion between colonies begins.

Five ramets of *Orbicella faveolata* from the same donor colony and of similar size were each fragmented into 0.86 ± 0.22 cm^2^ (average ± stdev) pieces and glued to 5 ceramic 20 × 20 cm tiles (Fig. 1A). Fragments were attached to tiles using cyanoacrylate gel and were spaced equidistant from one another, separated by approximately 1 cm. The number of fragments per tile ranged from 20 to 23. Similarly 5 separate individual donor colonies of *Pseudodiploria clivosa* (∼30 cm^2^) were fragmented into 3.05 ± 1.02 cm^2^ (average ± stdev) pieces and glued to 5 separate 20 × 20 cm tiles. Fragments were attached in the same way as above and spaced equidistant from one another, separated by approximately 1.5 cm with 9 fragments mounted to each tile. The tiles were placed in a shallow 340 liter raceway with flowing water drawn from a 24 m deep seawater well at a rate of 2.5 lpm. Temperature was maintained in the raceway between 22 and 26 °C by constant seawater turnover and four air stones (4 cm each) were used for water circulation and aeration. Algal growth was controlled by the shore snail *Batillaria minima*, daily siphoning of detritus, and manual removal of encroaching algae, particularly in the space between fragments. Additionally, live newly hatched *Artemia sp*. were broadcast in the raceway on a weekly basis. Top down photographs of each tile were taken at a fixed distance with a 1 cm cube used as a size reference on 9/2/2014, 12/1/2014, and weekly thereafter. One photo of *P. clivosa* was not taken on 1/19 and therefore was not included in the analysis. Area covered by live coral tissue was measured using these photos with Sigma Scan Pro 5. The tiles were followed for a period of 139 days.

### *Porites lobata* fusion experiment

**Figure 2:**
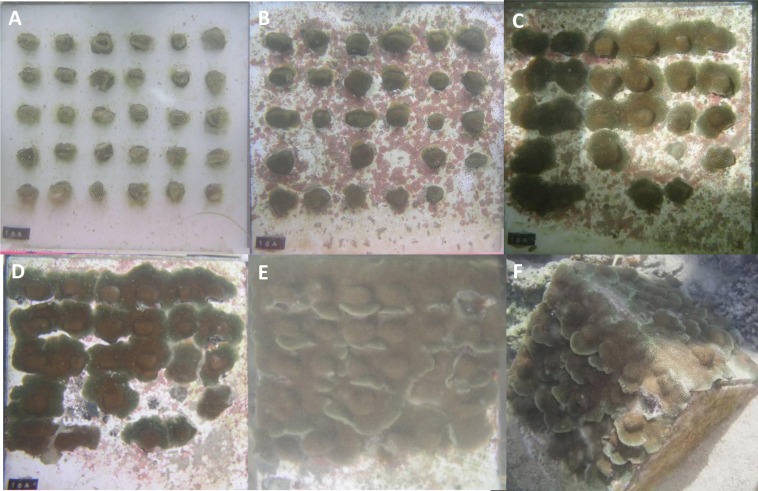
*Porites lobata* fragments fusing over ceramic tiles. (A) Thirty fragments were epoxied to ceramic tiles on 6/25/2006, yielding 23 cm^2^ of area covered by coral tissue; (B) after 38 days of growth, tissue begins to attach and 3 fragments are lost; (C) after 125 days of growth, tissue begins to come in contact with other colonies; (D) after 205 days of growth, most fragments are fused and area covered by tissue is 178 cm^2^; (E) after 368 days of growth the substrate is completely covered; (F) the resulting colony is approximately a half meter in diameter after one year.

*Porites lobata* fragments (*n* = 240 total fragments from a ca 15 cm portion of a single donor colony) were fragmented to 0.69 ± 0.33 cm^2^ (average ± stdev) and epoxied with approximately 2 cm of space between fragments to 30 × 30 cm glossy white ceramic tiles (30 fragments per tile) with marine epoxy (Splash Zone Compound; Woolsey/Z-spar Inc., Rockaway, New Jersey, USA; Fig. 2A). The tiles were mounted to triangular concrete bases. The eight tiles were divided into two tanks at Kewalo Marine Laboratory that had notable differences in both biotic and abiotic conditions. The ‘cleaned’ tank was exposed to full sun, while the ‘established’ tank was partially shaded. The ‘established’ tank had been continuously running for over 5 years as a mesocosm tank with sand, live rock, and a variety of fish and invertebrates, while the ‘cleaned’ tank was emptied and cleaned prior to the experiment and only a few snails (*Trochus inextus*) and urchins (*Tripneustes gratilla*) were added to control algal growth (these species were also present at similar densities in the ‘established’ tank). Hobo pendant light and temperature loggers (Onset Computer Corporation, Bourne, Massachusetts, USA) recorded data hourly for the duration of the experiment. Digital photographs were taken from a fixed photo frame with a scale, and top down area covered by live coral tissue. Tissue area was measured with ImageJ v 1.0 after fragmentation on 6/24/2006 and after 38 days of growth on 1/15/2007. Fragment tiles were kept in round fiberglass tanks (4 m diameter, 1 m deep) with unfiltered seawater from the same source, each receiving approximately 10 lpm. The fastest growing module (10a) was further monitored and photographed at 125, 205, and 368 days of growth, although after 205 days the area of each individual nubbin was no longer able to be measured since nearly all of the fragments had fused together.

### Additional observations

A *Montipora capitata* colony was fragmented and attached to ceramic tiles using the same method as for the *Porites lobata* fusion experiment. The colonies were photographed after 3 months growth and again after six years of growth (Figs. S1A–S1C). *Pocillopora meandrina* was attached to garden variety plastic mesh fencing material by cutting the mesh, then forcing the fragments between the rigid plastic tabs such that the fragment was secured (Figs. S1D–S1F), this method required no adhesive, is very fast, and also worked for *Porites compressa* (Figs. S2A and S2B). For *Porites astreoides*, 6 fragments were attached to live rock using cyanoacrylate gel and photographed after fragmentation and again after 706 days. Three fragments of *Solenastrea bournon*i were similarly attached and observed over a period of 511 days (Table 1).

**Table 1:** Rates of area increase for a variety of corals under a wide range of conditions.

Genus	Species	*n*	Start area (cm^2^)	End area (cm^2^)	Obs. period (days)	Rate (cm^2^/month)	% increase
*Orbicella*	*faveolata*	104	89.0	382.0	139	63.2	329
*Pocillopora*	*meandrina*	5	6.0	24.9	435	1.3	315
*Porites*	*lobata*	30	23.0	178.0	205	22.7	674
*Porites*	*lobata*	217	5.5	12.6	38	5.6	129
*Porites*	*astreoides*	6	26.2	139.0	706	4.8	431
*Pseudodiploria*	*clivosa*	45	136.0	345.0	132	47.5	154
*Solenastrea*	*bornouni*	3	3.9	7.9	511	0.2	103

## Results

The overall rate of growth for common Atlantic and Pacific corals across all observations was ∼20 cm^2^/month ± 25 cm^2^/month (average ± standard deviation), with a minimum of 0.2 cm^2^/month for *Solenastrea bournoni* and a maximum of 63.2 cm^2^/month for *Orbicella faveolata* (Table 1). These observations occurred over variable sampling periods and under various sampling conditions and some of these factors were examined in more detailed experiments for *Orbicella faveolata, Pseudodiploria clivosa,* and *Porites lobata*.

### *Orbicella faveolata* and *Pseudodiploria clivosa* fusion experiments

**Figure 3:**
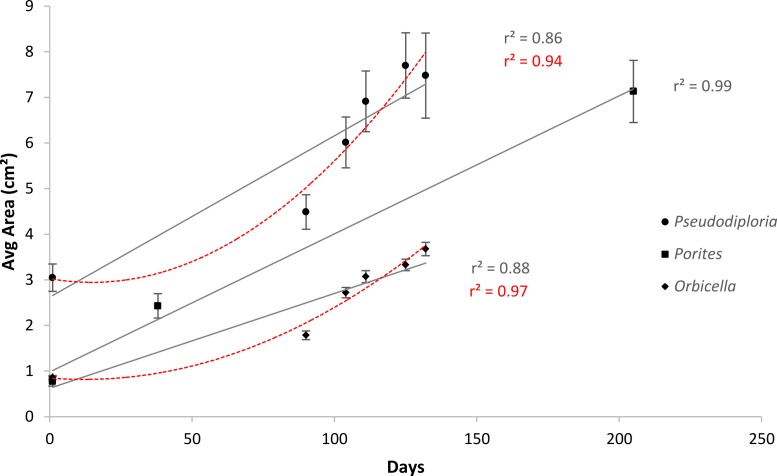
Average increase in coral area over ceramic tiles. Linear (gray lines) and polynomial (red lines) values of *Orbicella faveolata* (black diamonds) and *Pseudodiploria clivosa* (black circles) from 9/2/2014 to 1/19/2015, and *Porites lobata* (black squares) from 6/25/2006 to 1/17/2007.

**Figure 4:**
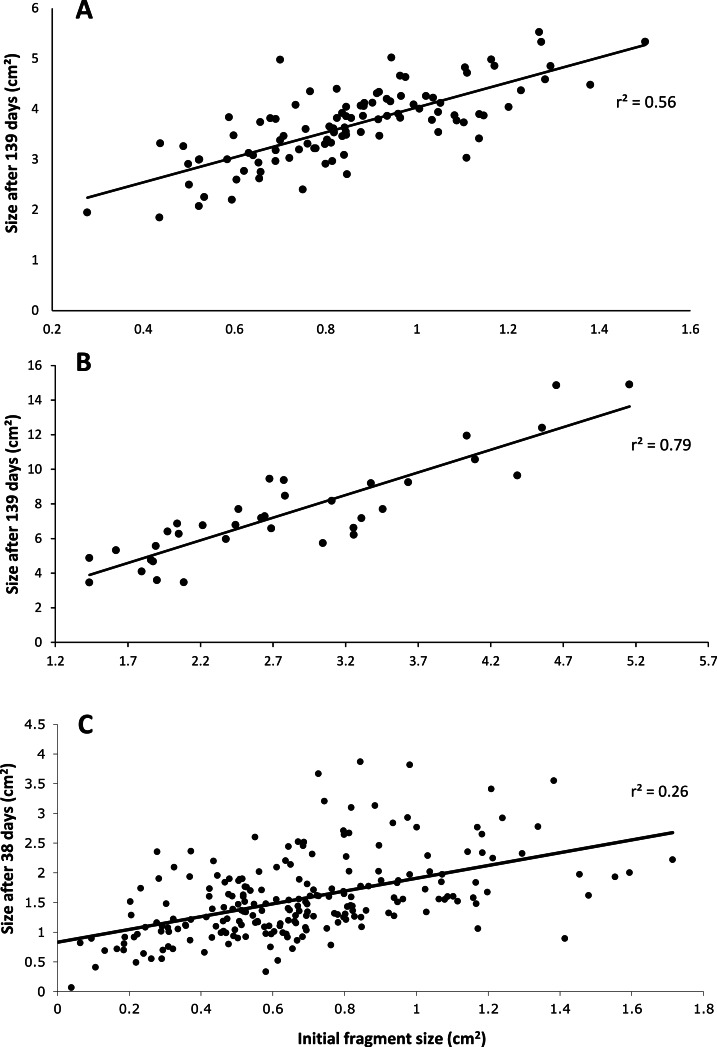
Relationship between initial size and final size. (A) Initial size of *Orbicella faveolata* versus size after 132 days of growth; (B) initial fragment size of *Pseudodiploria clivosa* versus 139 days of growth; (C) initial size of fragments of *Porites lobata* versus size after 38 days of growth.

After 139 days *O. faveolata* fragments increased in size by 329% with 13.5% of the fragmented colonies fusing together, while *P. clivosa* fragmented colonies increased in area by 154% with 31.1% of colonies fusing (Table 1 and Fig. 1). No fragments of either species detached or perished during the experiment. The growth rates of both species appeared to be approximately linear, explaining 86% of the variation for *P. clivosa*, and 88% for *O. faveolata* (Fig. 3). A second order polynomial regression explained 94% of the variance for *P. clivosa* and 97% of the variance for *O. faveolata* (Fig. 3). A linear regression between initial fragment size and final fragment size further indicated that growth rates are related to colony size, with larger fragments growing at a faster rate, explaining 56% of the variability in *O. faveolata* and 79% in *P. clivosa* (Fig. 4).

### *Porites lobata* fusion experiment

For *P. lobata*, the overall rate of increase was 5.6 cm^2^ per month; however the rates of tissue spreading differed according to tank, which contrast in both biotic and abiotic conditions. Module 10A had the highest rates of growth and was followed for additional time intervals after the tank comparison experiment. After 205 days, the rate of tissue increase was 22.7 cm^2^ per month, a 357% increase in area covered by tissue (Fig. 2). The majority of coral colonies were fused and it was no longer possible to measure the growth of individual colonies since the entire 0.6 m module became a single fused colony (Fig. 2F).

**Figure 5:**
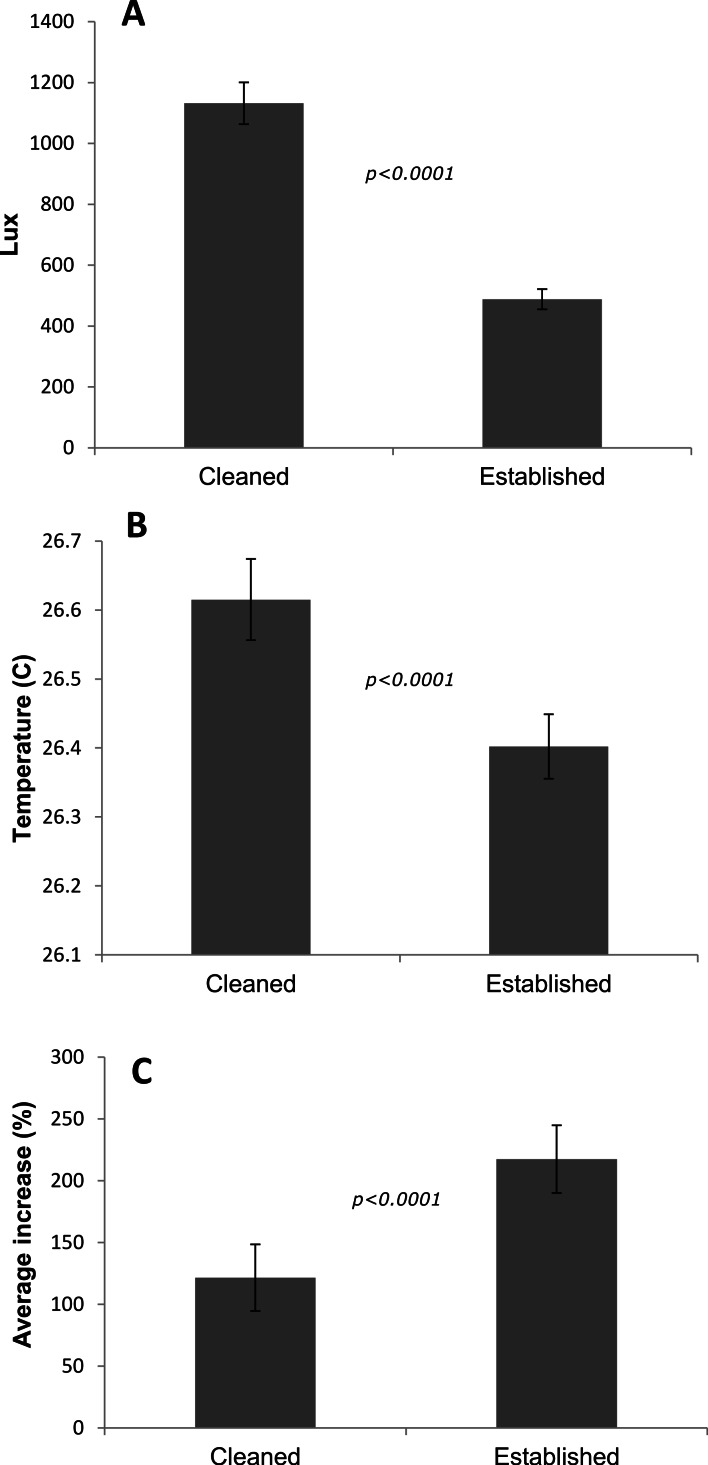
Differences between tanks for the *P. lobata* fusion experiment. (A) Irradiance; (B) temperature averages and 95% confidence intervals from loggers deployed during the *Porites lobata* fusion experiment; (C) average percent increase in area covered by coral tissue. Error bars represent 95% confidence intervals, significance values are from *t*-tests.

The ‘cleaned’ and ‘established’ tanks had significant differences in irradiance and temperature (Table 2 and Fig. 5). The irradiance values for the ‘cleaned’ tank were more than twice as high as the ‘established’ tank, while temperature values were significantly different, they only differed by a few tenths of a degree Celsius (Table 2 and Fig. 5). Corals in the ‘cleaned’ tank increased in average area covered by coral tissue by 122%, while the ‘established’ tank increased by 217% over 38 days. The ‘established’ tank had higher attachment failure (10.2% of the fragments were missing, compared to only 1 out of 109 fragments missing for the ‘cleaned’ tank), the missing fragments were most likely caused by sea urchin grazing, since grazing marks were observed near the colonies in the ‘established’ tank, and no grazing marks were observed in the ‘cleaned’ tank. The ‘established’ tank had red coralline algae covering a large proportion of the tiles after 38 days (Figs. 2B–2D), while the ‘cleaned’ tank had almost no coralline algae. The initial fragment size was related to the rate of growth, with a linear equation explaining nearly 26% of the variability, with 1 cm^2^ fragments nearly doubling in area over 38 days (Fig. 4).

**Table 2:** Survivorship, rates of growth and attachment failure for the *Porites lobata* fusion experiment. Averages ± 95% confidence intervals are presented, survivorship excludes missing colonies.

Tank	Lux	Temp C	*n*	Net growth (cm^2^)	Increase (%)	Missing (%)	Survival (%)
Cleaned	1,132 ± 96	26.62 ± 0.03	109	0.63 ± 0.07	122 ± 27	0.01	99
Established	488 ± 33	26.40 ± 0.03	108	1.21 ± 0.12	217 ± 27	10.2	92

## Discussion

### *Orbicella faveolata* and *Pseudodiploria clivosa* fusion experiments

In less than 4 months micro-fragments of *O. faveolata* increased a total of 293 cm^2^ while *P. clivosa* fragments grew 222 cm^2^. This corresponds to ∼11 cm and ∼9 cm of increased colony diameter respectively assuming circular colonies; however the present study measures changes in area covered by thin sheets of encrusted tissue, therefore these rates are not directly comparable to most field studies which measure change in maximum diameter or linear extension, for example, Caribbean corals grow at a rate of 0.5–1 cm per year (Hubbard & Scaturo, 1985; Logan, Yang & Tomascik, 1994; Cruz-Piñón, Carricart-Ganivet & Espinoza-Avalos, 2003). Nevertheless, starting with only 89 cm^2^ of *O. faveolata* tissue and 136 cm^2^ of *P. clivosa* tissue, four months of growth yielded a 329 and 154% increase in area respectively.

Growth rates for both species fit the expectations of linear rates of growth, explaining between 86 and 88% of the variance; however, a second order polynomial curve explained between 94 and 97% of the variation indicating that growth rates likely accelerated towards the end of the experiment (Fig. 3). The differences in growth rates through time could be due to a variety of factors; however the initial size of fragment is clearly important, with smaller sized fragments growing at a slower rate than larger fragments (Fig. 4). It was beyond the scope of this experiment to determine if rates of tissue spreading correspond to seasonality, temperature, colony age, or other biotic or abiotic factors; however, previous work has also found clear relationships between size and growth rates, with larger fragments growing at a faster rate (Edwards & Clark, 1999; Lirman et al., 2010; Lirman et al., 2014).

### *Porites lobata* fusion experiment

Similar to the *O. faveolata* and *P. clivosa* experiment, growth in terms of area covered by tissue for *P. lobata* is orders of magnitude higher than previously recorded, although it is important to emphasize that tissue spreading is not directly comparable to linear extension or volumetric growth. *P. lobata* grows on the order of ∼1 cm per year in linear extension or colony diameter, and these growth rates are known to be highly variable depending on environmental conditions (Lough & Barnes, 2000; Smith, Barshis & Birkeland, 2007; Elizalde-Rendón et al., 2010). This study documented growth of up to ∼22 cm^2^ per month (Table 1), which corresponds to ∼5 cm per month increase in diameter assuming a circular colony. These rates are similar to observations of the highest rates of healing from artificial lesions, which also varied considerably depending on environmental conditions (Van Woesik, 1998; Denis et al., 2011). Roff et al. (2014) documented extraordinary rates of tissue regrowth after a mass bleaching event in French Polynesia, from recolonization of *Porites* skeleton from surviving cryptic patches of live tissue, and similar large scale rapid recovery has also been observed for a coral community recovering from algal overgrowth (Diaz-Pulido et al., 2009). These combined observations indicate that the capacity for rapid tissue spreading may have been previously overlooked or underappreciated.

In the case of the *P. lobata* experiment, the differences in tissue spreading rates between tanks may be attributed to a variety of abiotic factors (e.g., temperature, or large difference in irradiance: Table 2), biotic factors (e.g., presence of CCA, algal biomass, bacterial diversity), or a combination of both. Since light levels were approximately twice as high in the cleaned vs. established tanks, and since photoinhibition can decrease growth rates, this may contribute to the significantly lower rates between treatments. The ‘established’ tank was a mesocosm that had at least 5 years to stabilize; providing grazing of herbivores to prevent algal blooms as well as likely increased diversity in terms of microbial, planktonic, and benthic micro-fauna. Newly established tanks on the other hand are inherently less stable and prone to monotypic blooms of microorganisms such as diatoms and ciliates and bacteria that may promote infection or inhibit growth. Another critical difference between treatments was the presence of crustose coralline algae in the ‘established’ tank, which is known to be important for coral settlement and growth (Bak, 1976; Harrington et al., 2004). Attachment failure was higher in the ‘established’ tank, however the higher rates of growth resulted in significantly higher growth overall. Although the differences in coral growth rates were significantly different in each tank, the potential causes could not be determined from this experiment; however, it is clear that growth rates are highly sensitive to various factors, which is fertile ground for further work towards the development of growth assays as well as for optimization of growth.

### Potential for reef restoration, growth assays, and coral aquaculture

As coral reefs are declining, there is increased demand for more responsible coastal development (Nyström, Folke & Moberg, 2000; Bellwood et al., 2004). There is also increased demand for sustainable sources of coral material for aquaculture, research, mitigation, and restoration projects (Rinkevich, 2008; Leal et al., 2014). The micro-fragmentation-fusion strategy effectively manipulates the surface area of a coral on to a two dimensional plane, over which small colonies rapidly spread tissue and fuse (Page, 2013). The ability to encrust coral onto a variety of substrates presents opportunities to design and test a variety of novel approaches for coral cultivation and transplantation such as mass production of small ‘seedlings,’ or larger modular designs. Covering a complex three dimensional structure with coral would effectively combine the benefits of coral transplantation with artificial reefs (Abelson, 2006), however detailed longer term studies are needed to determine the physiological and reproductive effects of the process, and to evaluate advantages over traditional direct transplantation, which typically results in small fragments that are prone to high rates of mortality (Yap, Alvarez & Dizon, 1998; Okubo, Taniguchi & Motokawa, 2005). This method could potentially be extended to much larger scales to provide more sustainable sources of coral material and to fill knowledge gaps for cultivation of ‘slow growing’ species. Integration with an *in-situ* or *ex-situ* nursery phase for example could provide source material at scales previously not possible (e.g., Amar & Rinkevich, 2007; Shafir, 2010).

**Figure 6:**
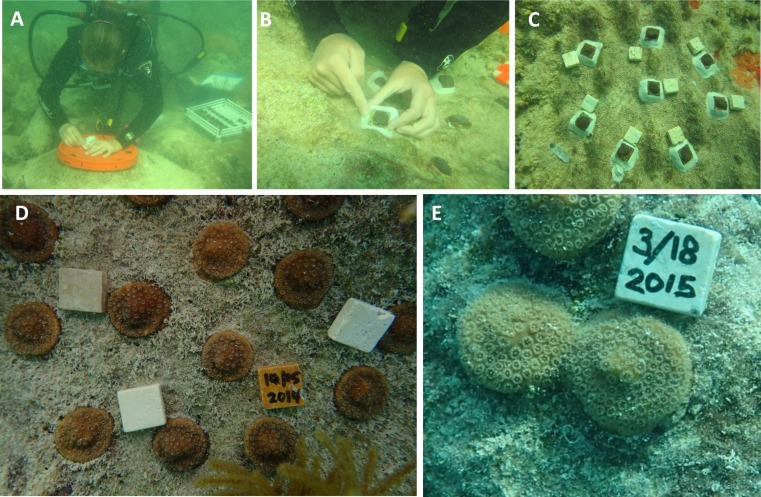
Example of using small nursery grown tiles for outplanting. (A) Substrate cleaning and site preparation; (B) tiles are mounted with epoxy; (C) finished array; (D) a separate array two months after outplanting; (E) an example from a separate array of colonies fusing and self-attaching in the field after five months of growth.

The ability of a coral fragment to grow onto the benthic substrate or ‘self-attach’ is critically important to the survival of the colony and the success of the transplantation effort (Guest et al., 2011). Here, we observe self-attachment by tissue spreading, over a wide variety of substrates (Figs. S1 and S2), which creates opportunities to improve transplantation, and furthermore the fusion method may be exploited to increase the chance of self-attachment by fusion over the benthic substrate (e.g., Figs. 6D and 6E). Field trials are currently underway to develop methods to efficiently encourage nursery grown coral to fuse and self-attach over the benthic substrate (Fig. 6). Experiments are currently underway to test the utility of this method for restoring *O. faveolata*, *M. cavernosa*, and *P. clivosa* to reefs that were affected by an anomalous cold temperatures occurring in early 2010 (Lirman et al., 2011). Three out-planting efforts have been recently initiated under various conditions such as varying by season, number of fragments planted per plot, and location (inshore vs. offshore). The method may prove particularly advantageous for massive stony corals with poor recruitment and or slow growth rates. The majority of transplantation and restoration projects have focused on fast growing “weedy” species which can rapidly increase coral cover (Edwards & Clark, 1999; Shaish et al., 2010); however, these species tend to be less resilient to long term disturbance (Loya et al., 2001; Van Woesik et al., 2012).

This study demonstrates that the micro fragmentation and fusion method can be used to rapidly cover a variety of substrates with coral tissue, providing fertile ground for further work. This study illustrates ‘proof of concept’ by focusing on isogenic colony fusion, examining fusion between ramets from only one genet per species. Further work is needed to compare multiple genotypes to determine genotypic effects on growth rates and to examine genotype by environment interactions in greater detail, as well as possible effects and tradeoffs of allogeneic fusion. In addition to possible reef restoration and coral aquaculture applications, this technique could also be useful for developing standardized growth assays that could be deployed in the field to monitor tissue growth or recession and mortality in a reasonable time frame (e.g., weeks or months as opposed to years). These deployable standardized growth assays could be used for determining if candidate sites are amenable for coral transplantation, or for determining the species and size specific effects of exposure to sediment plumes or other land based sources of pollution, or for laboratory based toxicological assays. The widespread use of the method for reef restoration however requires additional work to determine how the process can affect longer term survivorship, reproduction, and resilience to a variety of stressors.

## Supplemental Information

10.7717/peerj.1313/supp-1 Supplemental Information 1 Raw data tables

10.7717/peerj.1313/supp-2 Figure S1 Additional examples of tissue fusion (A) *Montipora capitata* fragments on ceramic tile modules; (B) the same modules after ∼6 months of growth; (C) the same modules after ∼6 years of growth; (D) *Pocillopora meandrina* fragments after fragmentation; (E) the *P. meandrina* fragments fusing over plastic mesh after several months; (F) the colony after 435 days.

10.7717/peerj.1313/supp-3 Figure S2 Examples of tissue spreading and fusion over plastic mesh (A) *Porites compressa* fragments spreading over plastic mesh after 5 months of growth; (B) two fragments of *Porites compressa* fusing after ∼4 months of growth on plastic mesh.
