# Supplementary figures and images for: Growing coral larger and faster: micro-colony-fusion as a strategy for accelerating coral cover
Source: PeerJ. 2015 Oct 20;3:e1313. doi: 10.7717/peerj.1313 (PMC4614846; doi:10.7717/peerj.1313)

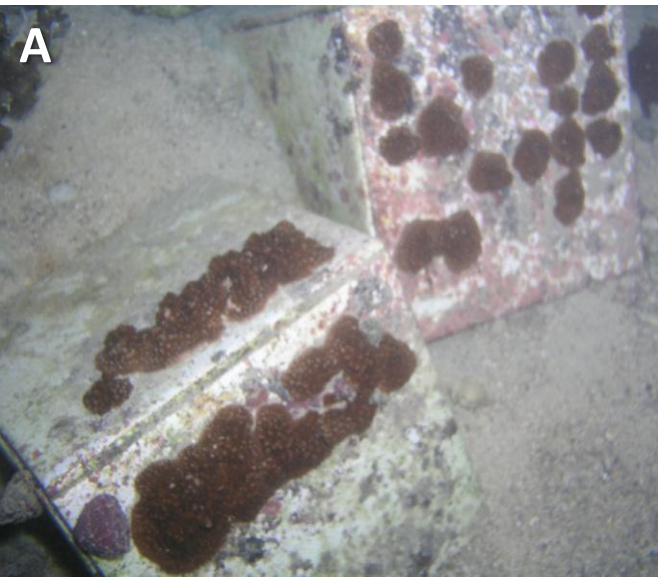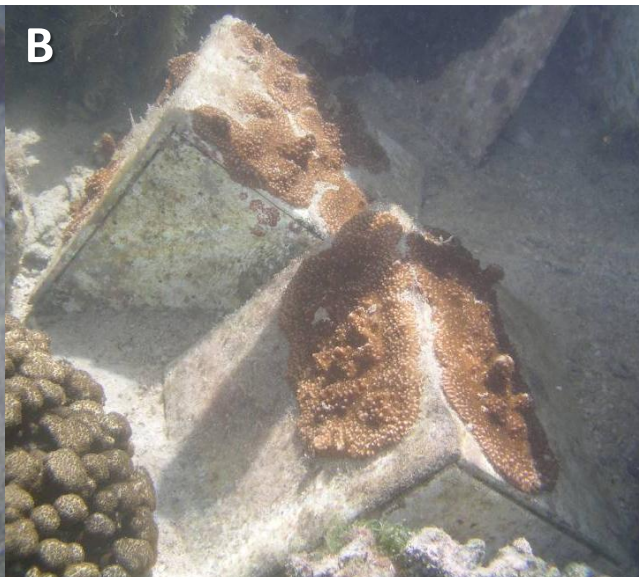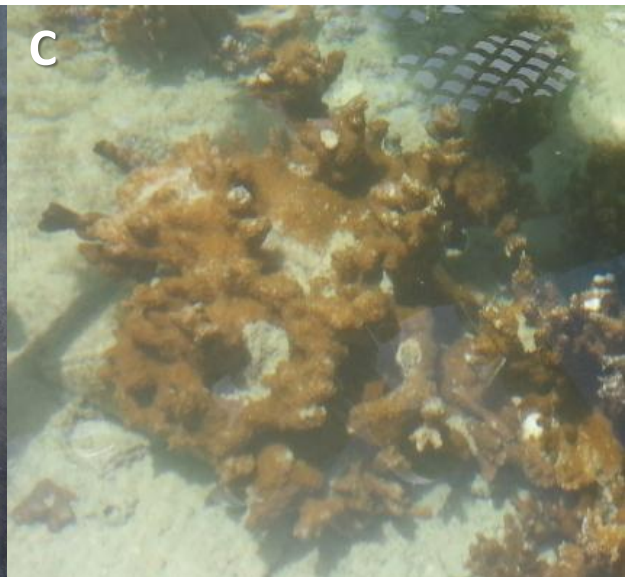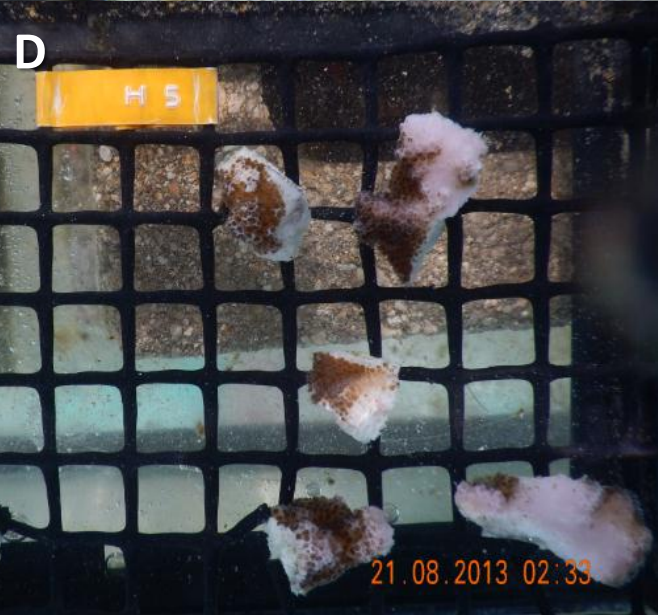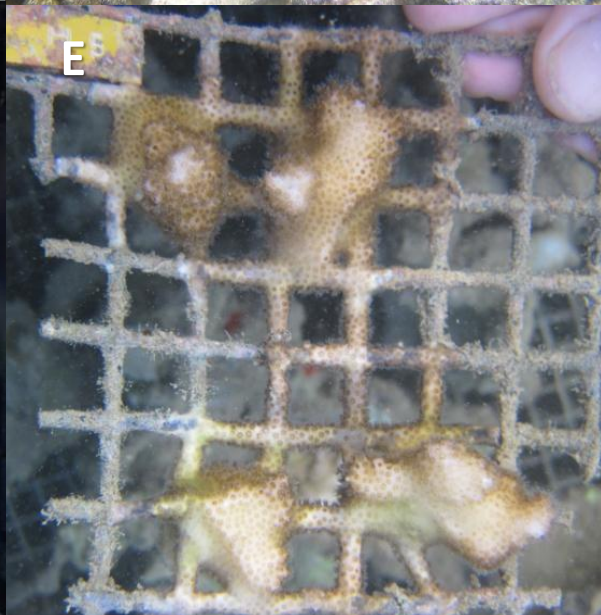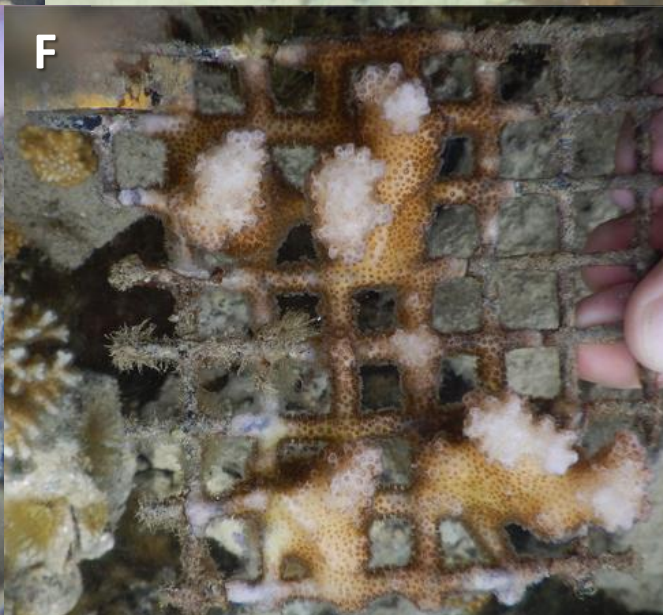

Supplement: Figure S1 — (A) Montipora capitata fragments on ceramic tile modules; (B) the same modules after ∼6 months of growth; (C) the same modules after ∼6 years of growth; (D) Pocillopora meandrina fragments after fragmentation; (E) the P. meandrina fragments fusing over plastic mesh after several months; (F) the colony after 435 days. [file peerj-03-1313-s002.pdf]

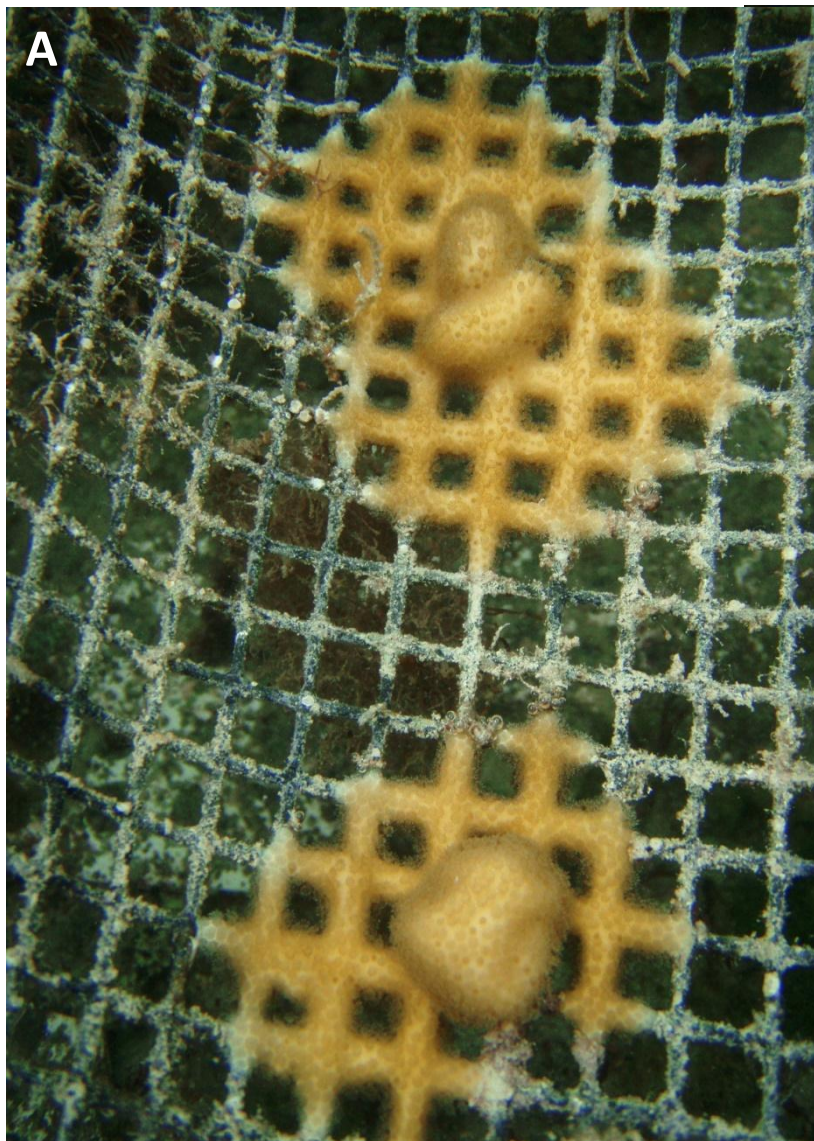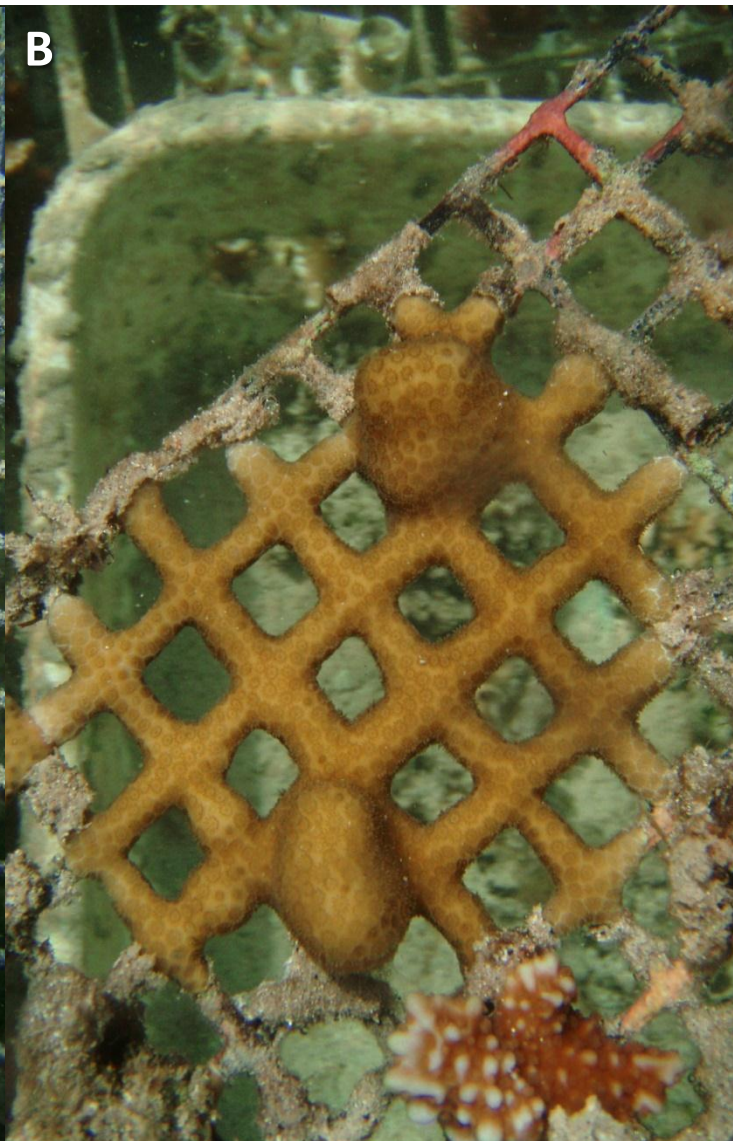

Supplement: Figure S2 — (A) Porites compressa fragments spreading over plastic mesh after 5 months of growth; (B) two fragments of Porites compressa fusing after ∼4 months of growth on plastic mesh. [file peerj-03-1313-s003.pdf]
